# GLP-1 Receptor Agonists and Diabetic Kidney Disease: A Call of Attention to Nephrologists

**DOI:** 10.3390/jcm9040947

**Published:** 2020-03-30

**Authors:** José Luis Górriz, María José Soler, Juan F. Navarro-González, Clara García-Carro, María Jesús Puchades, Luis D’Marco, Alberto Martínez Castelao, Beatriz Fernández-Fernández, Alberto Ortiz, Carmen Górriz-Zambrano, Jorge Navarro-Pérez, Juan José Gorgojo-Martinez

**Affiliations:** 1 Nephrology Department, Hospital Clínico Universitario, INCLIVA, Universidad de Valencia, 46010 Valencia, Spain; chuspuchades@gmail.com (M.J.P.); luisgerardodg@hotmail.com (L.D.); 2 Nephrology Department, Hospital Universitari Vall d’Hebron, Universitat Autònoma de Barcelona, 08035 Barcelona, Spain; mjsoler01@gmail.com (M.J.S.); clara.garcia@vhebron.net (C.G.-C.); 3 Unidad de Investigación y Servicio de Nefrología, Hospital Universitario Nuestra Señora de Candelaria, Santa Cruz de Tenerife, Universidad de La Laguna, 38200 Tenerife, Spain; jnavgon@gobiernodecanarias.org; 4 IIS-Fundación Jimenez Diaz UAM and School of Medicine, Universidad Autonoma de Madrid, 28040 Madrid, Spain; albertomcastelao@gmail.com (A.M.C.); aortiz@fjd.es (A.O.); 5 Nephrology Department, Bellvitge University Hospital, Hospitalet, 08907 Barcelona, Spain; beaff26@hotmail.com; 6 CAP Sant Pere, ABS Reus 1, 43202 Tarragona, Spain; carmengorrizz@gmail.com; 7 Hospital Clínico Universitario Valencia, INCLIVA, Universidad de Valencia, 46010 Valencia, Spain; jorgenavper@gmail.com; 8 Unidad de Endocrinología y Nutrición, Fundación Hospital Alcorcón, 28922 Madrid, Spain; juanjo.gorgojo@gmail.com

**Keywords:** chronic kidney disease, diabetic kidney disease, GLP-1

## Abstract

Type 2 diabetes mellitus (T2DM) represents the main cause of chronic kidney disease (CKD) and end-stage renal disease (ESKD), and diabetic kidney disease (DKD) is a major cause of morbidity and mortality in diabetes. Despite advances in the nephroprotective treatment of T2DM, DKD remains the most common complication, driving the need for renal replacement therapies (RRT) worldwide, and its incidence is increasing. Until recently, prevention of DKD progression was based around strict blood pressure (BP) control, using renin–angiotensin system blockers that simultaneously reduce BP and proteinuria, adequate glycemic control and control of cardiovascular risk factors. Glucagon-like peptide-1 receptor agonists (GLP-1RA) are a new class of anti-hyperglycemic drugs shown to improve cardiovascular and renal events in DKD. In this regard, GLP-1RA offer the potential for adequate glycemic control in multiple stages of DKD without an increased risk of hypoglycemia, preventing the onset of macroalbuminuria and slowing the decline of glomerular filtration rate (GFR) in diabetic patients, also bringing additional benefit in weight reduction, cardiovascular and other kidney outcomes. Results from ongoing trials are pending to assess the impact of GLP-1RA treatments on primary kidney endpoints in DKD.

## 1. Introduction

Chronic kidney disease (CKD) is among the most common complications of type 2 diabetes mellitus (T2DM). In several contemporary studies, 28% to 43% of patients with T2DM have CKD, defined as either a glomerular filtration rate (GFR) below 60 mL/min/1.73 m^2^ or a urinary albumin to creatinine ratio (UACR) >30 mg/g [1,2]. In pivotal studies such as UKPDS (UK Prospective Diabetes Study), 25% of T2DM patients developed microalbuminuria within 10 years of diagnosis. Relatively fewer patients develop macroalbuminuria, but in those who do, the death rate is higher than the rate of progression to more advanced nephropathy. Of note, the low rate of renal replacement therapy (RRT) observed likely reflects the high mortality of patients with diabetes-associated CKD, which may reach up to 20% per year [3,4].

Diabetic kidney disease (DKD) is a leading cause of morbidity and mortality in diabetes. CKD is associated with all-cause and cardiovascular mortality in patients with diabetes. Indeed, the excess mortality of diabetes occurs mainly in individuals with diabetes and proteinuria and stems not only from end-stage renal disease (ESRD) but also from cardiovascular disease. As such, DKD is on track to surpass all other diabetic complications in terms of attributable morbidity and mortality [4]. Indeed, on a global level, CKD is expected to become the fifth cause of death worldwide within 20 years, and diabetes-associated CKD is a key contributor to the increasing prevalence of CKD [5].

In recent years it has become apparent that diabetes-associated CKD is more heterogeneous than previously thought, and may consist predominantly of tubular, interstitial and/or vascular involvement rather than glomerular injury. Thus, patients with decreased GFR may not have pathological albuminuria, and the term DKD is now preferred, leaving the term diabetic nephropathy for histologically confirmed lesions [6]. Despite advances in the nephroprotective treatment of T2DM, DKD remains the most common complication, and its incidence keeps increasing, driving the need for RRT worldwide [6,7]. It is, therefore, vital to optimize risk stratification and therapeutic strategies for both DM2 and DKD patients in order to decrease associated morbimortality, mainly from cardiovascular disease, and the need for RRT. Our review will focus on the potential role of glucagon-like peptide-1 receptor agonists (GLP-1RA) as nephroprotective agents in T2DM. 

## 2. Management of Diabetic Kidney Disease

Until recently, prevention of DKD progression was based on strict blood pressure (BP) control, using renin–angiotensin system blockers that simultaneously reduce BP and proteinuria, adequate glycemic control and control of cardiovascular risk factors (dyslipidemia, obesity and smoking), as well as nephrotoxic drug avoidance [6,8,9] (Table 1). This approach nonetheless results in significant residual renal and cardiovascular risk.

### 2.1. BP Control

BP should be controlled with drugs that reduce cardiovascular events (angiotensin-converting enzyme inhibitors, angiotensin II receptor blockers, thiazide-like diuretics or calcium channel blockers) [8]. However, despite the controversy surrounding target BP levels in T2DM patients, over 50% have BP <140/90 mmHg, but only around 20% have BP <130/80 mmHg [9].

### 2.2. Blood Glucose Control

Optimizing glucose control in DKD patients is challenging due to an increased risk of adverse effects from certain glucose-lowering agents. Both UKPDS and DCCT have clearly demonstrated that progression of retinopathy and nephropathy is linked to glycemic control and it is crucial that patients maintain HbA1c levels less than or equal to 6.5% to minimize disease progression [10]. Nevertheless, the drugs previously available to reach this goal were associated with a high risk of hypoglycemia. In the last few decades, therefore, blood glucose objectives have become more moderate, which has undoubtedly had an impact on microvascular complications, including nephropathy. Accumulation of kidney-excreted drugs, especially sulfonylureas and insulin, may result in repeated hypoglycemic episodes [11], while metformin may cause potentially lethal lactic acidosis [12,13]. Some drugs that can be administered despite low eGFR values are marred by side effects such as hypoglycemia with repaglinide, volume retention and risk of distal fractures with pioglitazone, or may impact negatively on CKD progression (e.g., dipeptidyl peptidase 4 inhibitors).

Among the agents used to treat hyperglycemia, only sodium–glucose co-transporter type 2 inhibitors (SGLT2i) and certain GLP-1RA have been shown to improve kidney outcomes regardless of glycemic control [14]. SGLT2i decreased both primary and secondary kidney endpoints in patients with DKD or at high cardiovascular risk, respectively [15,16,17]. These endpoints include progressive loss of GFR, new-onset albuminuria and RRT initiation, alone or in combination. However, the use of SGLT2i is still limited by the high cost and regulatory contraindications for patients with eGFR <60 mL/min/1.73 m^2^. It should be noted that this limit is driven by the perceived lack of benefit on glucose control at these low eGFR levels, since the mechanism of antihyperglycemic action depends on glycosuria. Nevertheless, recent trials demonstrated nephroprotection in patients with GFR >30 mL/min/1.73 m^2^, even when the drug was maintained until the initiation of dialysis [16,18]. Some GLP-1RA, including liraglutide [19], semaglutide [20] and dulaglutide [21], have also shown renal benefits, and contrary to SGLT2i, can currently be administered up to an eGFR of 15 mL/min/1.73 m^2^.

### 2.3. Obesity

The prevalence of obesity and abdominal obesity in T2DM patients remains high. In Spain, the country projected to have the longest life expectancy by 2040, this prevalence is 50% and 68%, respectively [5,22]. Obesity and overweight are known renal progression factors, although most studies on the renoprotective impact of weight loss are retrospective or are prospective but with only one-arm observational studies. In obese patients (with and without diabetes), weight loss induces a significant reduction in proteinuria [23], which is observed rapidly after weight loss and correlates with weight reduction. Similarly, the correction of metabolic syndrome components along with weight reduction has been associated with slower DKD progression [24].

In T2DM patients with obesity and advanced CKD (GFR < 40 mL/min/1.73 m^2^ and albuminuria > 30 mg/g), the combination of a hypocaloric diet and physical exercise was associated with 12% weight and 36% albuminuria reduction, accompanied by improved renal function and glycemic control [25]. This benefit was recently demonstrated in the SOS study (Swedish Obese Subjects). This study compared 2010 patients undergoing bariatric surgery (17% with T2DM) with 2037 obese controls (13% with T2DM) who received standard care for obesity and an average follow-up of 18 years. Patients undergoing bariatric surgery had a 67% reduction in the incidence of renal endpoint (CKD stage 4–5, dialysis or transplant) compared to controls (*p* < 0.001). The beneficial impact was more evident in patients with pathological albuminuria [26].

## 3. GLP-1 Receptor Agonists

The incretin effect describes the phenomenon whereby, in healthy individuals, oral glucose elicits higher insulin secretory responses than intravenous glucose, despite inducing similar levels of glycemia. This effect, which is uniformly defective in T2DM patients, is mediated by the gut-derived incretin hormones glucose-dependent insulinotropic polypeptide (GIP) and glucagon-like peptide-1 (GLP-1). Two incretin hormones have been identified produced by entero-endocrine K cells, whereas GLP-1 is mainly secreted from L cells located throughout the intestine, more abundantly towards the distal ileum and colon. GLP-1 acts through binding to its receptor (GLP-1R) triggering a downstream signaling cascade. GLP-1R is a class B G protein-coupled receptor not only expressed in the pancreas and central nervous system, but also detected in lower levels in the gut, kidneys, lungs, liver, heart, muscle, peripheral nervous system and other tissues. GLP-1 increases insulin secretion in response to nutrients, particularly glucose (the so-called incretin effect) and suppresses glucagon secretion from pancreatic islet cells, with a reduction in postprandial glucose levels as the net result [27]. 

GLP-1 seems also to play a role in the central regulation of feeding by increasing satiety signals and reducing appetite, resulting in decreased food intake and subsequent weight loss. Further, GLP-1 exerts effects on the gastrointestinal tract by slowing the gastric emptying rate and small intestine peristalsis, which conditions slower absorption of glucose. Depending on the molecule (native or recombinant long-acting) and administration route, GLP-1RA have broad pleiotropic action on metabolism. All these actions are transduced by a single GLP-1R located in many organs including the kidney. Among the numerous beneficial effects mediated by GLP-1RA are blood glucose regulation, body weight reduction due to food intake inhibition and reduced gastric motility, cell proliferation stimulation, inflammation and apoptosis reduction, and improved cardiovascular function, neuroprotection and renoprotection [28,29]. 

In T2DM individuals, circulating GLP-1 levels are similar to those found in normoglycemic individuals, yet partial resistance to the insulinotropic effects of GLP-1RA is seen in some T2DM patients at physiological and pharmacological concentrations.

GLP-1 response to oral glucose tolerance test is up to 25% lower in individuals with prediabetes or T2DM than in those with normal glucose regulation. Whether a defective incretin system in T2DM is caused by decreased responsiveness of β cells to GLP-1 and glucose-dependent insulinotropic polypeptide (GIP), or by hyposecretion of incretin hormones, remains unclear. Importantly, in T2DM the insulinotropic response to exogenous GIP administration is completely lost, while a partially preserved, substantial dose-dependent response to GLP-1 is observed [30]. 

Diminished insulin secretion in response to treatment with GLP-1RA has been associated with genetic and metabolic alterations, thus implicating genetic variation in the transcription factor 7-like 2 (TCF7L2), the loci for GLP-1R, wolfram syndrome 1 and chymotrypsinogen B1/2 [31]. Impaired proinsulin conversion could explain the mechanism for TCF7L2-associated diminished GLP-1RA efficacy as well as dependent repression of GLP-1R expression on b-cells [32]. Incretin action may also be reduced during hyperglycemia and also in some individuals with prediabetes, diabetes and insulin resistance [28]. Native GLP-1 has a very short half-life (about two minutes) because of rapid degradation by the endogenous enzymes dipeptidyl peptidase (DPP-4) and neutral endopeptidase.

GLP-1RA are a pharmacological family of peptides that stimulate the human GLP-1 receptor. They can be classified according to different characteristics, such as molecular size, chemical structure and duration of action. Based on their chemical structure, GLP-1 RA can be divided into two groups: incretin-mimetics (exendin-4 analogs) and human GLP-1RA. Incretin-mimetics are derived from exendin-4, a 39 amino acid peptide isolated from the saliva of the giant lizard Gila monster (Heloderma suspectum), and they share a 53% homology with human GLP-1. Such structural differences with human GLP-1 confer both exendin-4 and incretin-mimetics with resistance to inactivation by DPP-4. Currently, daily exenatide, once-weekly exenatide and lixisenatide are approved exendin-4 analogs, and efpeglenatide is an investigational drug in Phase 3 clinical development. Incretin-mimetic drugs are mainly eliminated by glomerular filtration, tubular reabsorption and subsequent proteolytic degradation, so their clearance is reduced in patients with renal insufficiency. Due to their partial homology to human GLP-1, these drugs may induce an immune response in some individuals; approximately 2–3% of patients develop inactivating antibodies. Human GLP-1 analogs show a close structural homology to native GLP-1, and they increase their half-life through several molecular modifications, such as attachment of fatty acid (liraglutide and semaglutide), albumin (albiglutide) or a constant fraction of immunoglobulin G4 (dulaglutide). Single substitutions of amino acids in the molecular sequence of native GLP-1 confer human GLP-1RA resistance to DPP-4 cleavage. Liraglutide, dulaglutide and semaglutide (subcutaneous and oral) are currently approved human GLP-1RA, since albiglutide was withdrawn from the market for commercial reasons. Human GLP-1RA are metabolized in target tissues via the common proteolytic pathway of large proteins, without a specific organ identified as the main route of elimination. They are protected from renal clearance by either their large molecular size or their noncovalent attachment to albumin. Due to their structural similarity to native human GLP-1, their immunogenicity is low [33]. 

Large-sized GLP-1RA, such as dulaglutide and albiglutide, are bound to large proteins, which makes it difficult for them to cross the blood–brain barrier and reach satiety centers. Therefore, a lower effect on body weight has been seen in clinical trials with these molecules in comparison to small-sized GLP-1 RA such as liraglutide or semaglutide [33]. 

Classification of GLP-1 RA into short- and long-acting agonists may be more useful from a practical viewpoint. Long-acting GLP-1 RA, such as liraglutide, dulaglutide, once-weekly exenatide or semaglutide, induce prolonged stimulation of the GLP-1R, leading to a greater reduction of fasting plasma glucose and HbA1c. Short-acting GLP-1 RA, such as daily exenatide or lixisenatide, show a lower effect on fasting plasma glucose, basal insulin secretion and HbA1c but induce a more pronounced decrease in postprandial glycemia. The effect of short-acting GLP1-RA on postprandial glycemia seems to be a consequence of delayed gastric emptying. In contrast, the effect of long-acting GLP-1 RA on gastric emptying is lost after a few weeks of treatment due to a tachyphylaxis mechanism [33].

Human GLP-1RA (liraglutide, semaglutide, albiglutide, dulaglutide) have been shown to reduce cardiovascular morbidity and mortality. In contrast, incretin-mimetics (daily exenatide, once-weekly exenatide, lixisenatide) have not demonstrated superiority in cardiovascular outcomes trials. Some experts argue that differences in study design and population, statistical power, adherence, withdrawals or even mere chance may explain these findings [33]. However, certain molecular differences will probably have contributed, at least partially, to the results:

- Immunogenicity. Exendin-4 analogs are immunogenic, and, in some patients, the drug is inactivated by antibodies, whereas human GLP-1RA rarely induce antibody formation.
- Tolerance. Exendin-4 analogs are metabolized and eliminated by the kidneys, so they accumulate in CKD patients (precisely those with the highest cardiovascular risk), which may favor their withdrawal due to gastrointestinal intolerance. Human GLP-1RA, in contrast, are not eliminated by the kidneys.
- Production of GLP-_19-36_. Exendin-4 analogs are fully resistant to inactivation by DPP-4. Conversely, human GLP-1RA may be partially metabolized to small amounts of the metabolite GLP-_19-36_, which could have an additional cardioprotective effect, acting in the endothelial mitochondria through a non-GLP-1 receptor pathway.

From a pharmacological point of view, GLP-1RA effects are mediated by their target molecule (GLP-1R). In this regard, Takayanagi et al. reported that target molecular occupancy could be a useful parameter for evaluating the clinical efficacy of these drugs [34]. They showed that GLP-1RA produce their clinical effect at a relatively low level of GLP-1R occupancy, suggesting that this parameter (GLP-1R occupancy rate) could be used to evaluate clinical efficacy irrespective of the drugs used. Moreover, applied to a single patient, it would be possible to evaluate the clinical efficacy of these drugs individually to make optimal treatment choices.

## 4. GLP-1RA in Diabetic Kidney Disease Patients

### 4.1. Pharmacokinetics

The structural difference in human GLP-1 confers exendin-4 and, by extension, incretin-mimetics, resistance to inactivation by DPP-4. Incretin-mimetics are eliminated mainly by glomerular filtration, tubular reabsorption and subsequent proteolytic degradation, so their clearance is reduced in patients with renal insufficiency. In contrast, GLP-1 analogs are metabolized locally in the target tissues by the common route of large proteins, without a specific organ identified as the main route of elimination. They are protected from renal clearance by their large molecular size or by their non-covalent binding to albumin [33,35]. 

A pharmacokinetic study with the administration of a single subcutaneous dose of semaglutide 0.5 mg showed that semaglutide exposure was similar between subjects with mild/moderate renal impairment or end-stage renal disease and subjects with normal renal function. This equivalence was not demonstrated in subjects with severe renal impairment, in which mean exposure was 22% higher [36]. 

**Figure 1:**
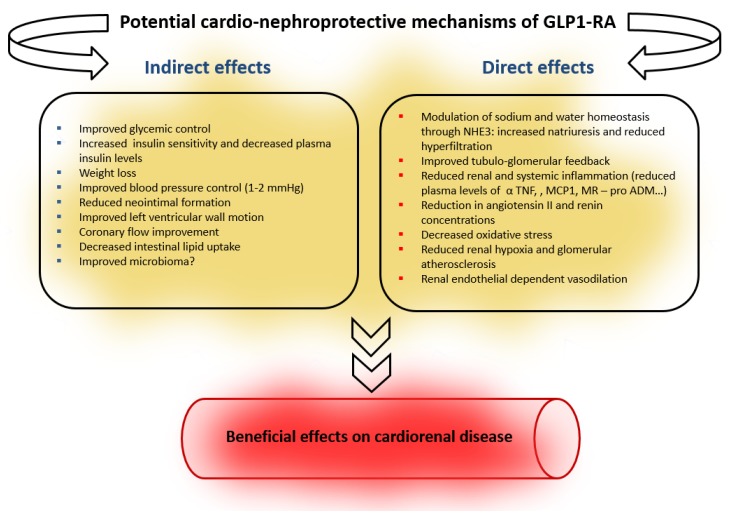
Potential mechanisms for the cardio-nephroprotective effect of GLP-1RA (modified from: Thomas [39] and Greco [40].

Thus, the European Medicines Agency (EMA) has approved the use of all commercially available human GLP-1 analogs up to eGFR of 15 mL/min/1.73 m^2^, while all exendin-4 analogs are contraindicated below 30 mL/min/1.73 m^2^, given the risk of accumulation and toxicity [20,37]. GLP-1RA may exert a beneficial action on the kidneys through blood glucose and BP-lowering effects, reduction of insulin levels and weight loss as well as possible direct cardio-nephroprotective mechanisms through actions on endothelial dysfunction and inflammation [38] (Figure 1).

### 4.2. Antihyperglycemic Efficacy of GLP-1RA in Patients with DKD

The range of antihyperglycemic medications in patients with advanced DKD is limited. When GFR falls below 30 mL/min/1.73 m^2^, patients are traditionally switched to insulin, metaglinides or DPP4i. Insulin treatment is associated with an increased risk of hypoglycemia [11], weight gain and increased sodium reabsorption in the proximal tubule [41]. DPP4i are modestly effective in reducing glycemia in all ranges of CKD, even in dialysis [42]. However, no cardiovascular or renal benefit has been observed [43], and even saxagliptin was associated with an increased incidence of heart failure [17]. By contrast, SGLT2i and some GLP-1RA were associated with improved cardiorenal outcomes. SGLT2i reduced cardiorenal risk in patients at high cardiovascular risk and in patients with DKD, as recently reported [44,45]. Cardiovascular and renal benefit is present despite only a modest reduction in weight and regardless of glycemic control. 

The GLP-1RA liraglutide [19,46], dulaglutide [37] and subcutaneous and oral semaglutide [20,47] have shown to improve glycemic control in patients with diabetes and very low eGFR, including dialysis patients for liraglutide [48]. In T2DM patients with moderate renal impairment, liraglutide demonstrated better glycemic control and weight reduction, with a good safety profile in terms of low hypoglycemia risk compared to placebo [49]. In addition, the AWARD-7 trial was able to demonstrate that when both were combined with insulin lispro, once-weekly dulaglutide attenuated eGFR decline more than titrated daily insulin glargine in T2DM and moderate-to-severe CKD patients. Note that these benefits were observed lowering HbA1c to a similar extent as insulin glargine, but with additional weight loss and the benefit of a lower rate of hypoglycemia in the weekly dulaglutide group as compared with daily insulin glargine, thus demonstrating increased safety [22]. In concordance with these findings, in the PIONEER 5 trial once-daily oral semaglutide 14 mg was superior to placebo in decreasing HbA_1c_ and body weight in patients with T2DM and moderate renal impairment, revealing a good safety profile, including renal safety and few hypoglycemic episodes [50]. 

## 5. GLP-1 Receptor Agonists and Cardiovascular Outcomes

**Figure 2:**
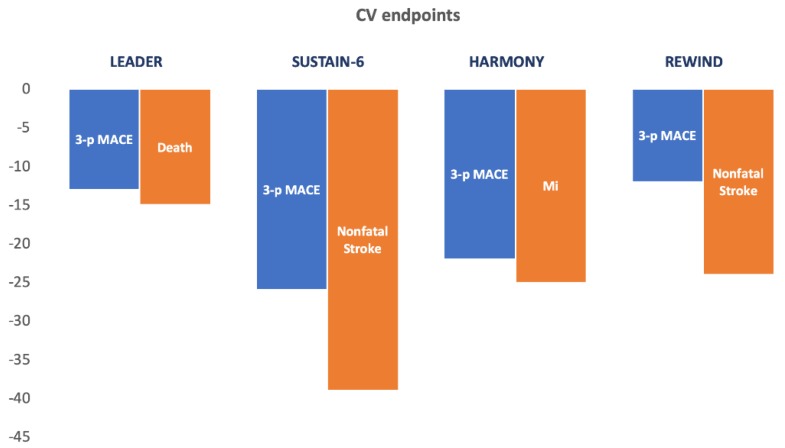
GLP-1RA RCT studies with known beneficial cardiovascular endpoints. Blue bars—percentage of 3-point MACE reduction with the intervention drug. Orange bars—percentage of individual primary cardiovascular endpoint reduction with the intervention drug. Three-point MACE, 3-point MACE; MI, fatal and nonfatal myocardial infarction.

The efficacy and safety of GLP-1RA has been evaluated in eight clinical trials with cardiovascular outcomes in 60,090 T2DM patients: ELIXA (lixisenatide) [50], EXSCEL (exenatide) [51], LEADER (liraglutide) [19,52], SUSTAIN 6 (subcutaneous semaglutide) [20], HARMONY (albiglutide) [53], REWIND (dulaglutide) [37], PIONEER 6 (oral semaglutide) [54] and FREEDOM-CVO (ITCA 650, a novel drug device delivering continuous exenatide) [55] (See Table 2, Figure 2).

The first reported trial, ELIXA, conducted in 6068 patients with a history of recent acute coronary syndrome and T2DM, was CV neutral, confirming the noninferiority of lixisenatide for three-point MACE [50]. The second one, LEADER, was conducted in 9340 T2DM patients with high cardiovascular risk and demonstrated both CV noninferiority and statistical superiority of once-daily treatment with liraglutide. The reduction in three-point MACE (HR 0.87 (95% CI 0.78–0.97)) with liraglutide was driven mainly by a reduction in CV death (HR 0.78 (95% CI 0.66–0.93)) [52]. The SUSTAIN 6 trial studied the effect of once-weekly treatment with 0.5 or 1 mg of long-acting semaglutide in 3297 T2DM patients at high risk for CV risk, defined by age 50 or older with established cardiovascular disease, chronic heart failure (New York Heart Association class II or III), or CKD stage 3 or higher or age 60 or older with at least one cardiovascular risk factor [20]. This Phase 3 trial demonstrated a favorable effect on three-point MACE accompanied by a significant decrease in nonfatal stroke (HR 0.61 (95% CI 0.38–0.99), *p* = 0.04). EXSCEL was the largest study performed in a usual-care setting including 14,752 T2DM patients with or without previous CVD. This study was CV neutral, confirming the noninferiority of once-weekly treatment with 2 mg long-acting extended-release exenatide [51]. The HARMONY trial studied the effect of once-weekly treatment with 30 or 50 mg long-acting albiglutide in 9469 T2DM patients with cardiovascular disease [53]. This trial demonstrated a favorable effect on three-point MACE accompanied by a significant decrease in myocardial infarction (HR 0.75 (96% CI 0.61–0.90), *p* = 0.003) [53]. PIONEER 6 studied the effect of once-daily oral semaglutide treatment with a target dose of 14 mg in 3183 T2DM patients aged 50 or older with established cardiovascular disease or CKD or aged 60 or older with cardiovascular risk factors alone [54]. This trial demonstrated the noninferiority of oral semaglutide for three-point MACE, with a non-significant 21% reduction in MACE in the treatment group [54]. The last published trial, REWIND, was conducted in 9901 T2DM patients with either a previous cardiovascular event or cardiovascular risk factors and demonstrated CV noninferiority and statistical superiority of once-weekly treatment with 1.5 mg dulaglutide. Dulaglutide reduced cardiovascular outcomes in both men and women with or without previous CV disease. The reduction in three-point MACE (HR 0.88 (95% CI 0.79–0.99)) with dulaglutide was mainly driven by a reduction in nonfatal stroke (HR 0.76 (95% CI 0.61–0.95)) [37]. 

The FREEDOM-CVO trial, designed to test CV safety in a pre-approval setting, evaluated the continuous delivery of exenatide in more than 4000 T2DM patients with CV disease. Although the final results have not yet been published, early on in May 2016, the company press release reported that the study had achieved all its clinical endpoints and was completed on time, confirming noninferiority in terms of CV outcomes [55].

A recently published systematic review and meta-analysis of cardiovascular outcome trials with GLP-1RA screened 27 publications and seven of the above-mentioned clinical trials [56]. Overall, GLP-1 receptor agonist treatment reduced MACE by 12% (HR 0.88, (95% CI 0.82–0.94); *p* < 0.0001). HRs were 0.88 (95% CI 0.81–0.96; *p* = 0.003) for death from cardiovascular causes, 0.84 (0.76–0.93; *p* < 0.0001) for fatal or nonfatal stroke and 0.91 (0.84–1.00; *p* = 0.043) for fatal or nonfatal myocardial infarction. GLP-1 receptor agonist treatment reduced all-cause mortality by 12% (HR 0.88, (95% IC 0.83–0.95; *p* = 0.001)), hospital admission for heart failure by 9% (HR 0.91, (95% IC 0.83–0.99; *p* = 0.028)), without increasing the risk of severe hypoglycemia, pancreatitis or pancreatic cancer [56].

Overall, the clinical trials mentioned in this section demonstrated noninferiority and CV neutrality for exendin-4 GLP-1RA, and cardiovascular protection for human GLP-1RA. This observed differential effect may be ascribed to different causes such as clinical trial design, studied population characteristics, and molecular differences between exendin-4 and human GLP-1RA.

All these data prompt the question of whether GLP-1RA has a class effect. Human GLP-1 analogs (liraglutide, semaglutide, albiglutide, predictably dulaglutide) have demonstrated CV morbidity and mortality reduction. In contrast, exendin-4 or incretin-mimetic analogs (exenatide, exenatide LAR, lixisenatide) have not achieved superiority in CV safety studies, a fact that points towards a possible class effect in this group of drugs. Other explanations for these differences include the study designs, populations studied, potency, withdrawals or even mere chance. However, certain molecular differences are likely to have contributed at least partially to these results, which supports the hypothesis of a class effect in GLP-1RA.

## 6. GLP-1 Receptor Agonists on Kidney Outcomes

**Figure 3:**
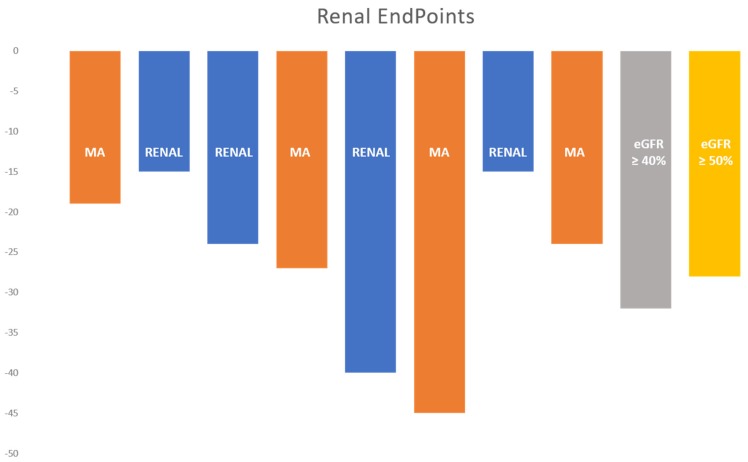
GLP-1RA RCT studies with known beneficial renal endpoints. Blue bars—percentage of composite renal endpoint reduction with the intervention drug. Orange bars—percentage of individual renal endpoint reduction with the intervention drug. Renal, combined renal endpoint; MA, macroalbuminuria; ↓ eGFR ≥ 40%, Sustained decline in eGFR of ≥40%; ↓ eGFR ≥ 50%, Sustained decline in eGFR of ≥50%.

To date, there are no published GLP-1RA trials with a primary endpoint of kidney events or enrolling only DKD patients. For this reason, insights into the renal impact of GLP-1RA were provided by cardiovascular outcomes trials (Table 3, Figure 3).

In the ELIXA (Evaluation of Lixisenatide in Acute Coronary Syndrome) trial [50,57], eGFR was <60 mL/min/1.73 m^2^ in 25% of lixisenatide and in 22% of placebo patients. In an exploratory analysis, lixisenatide decreased the percentage of albuminuria change compared to baseline when tested against placebo, although after adjusting for various variables, this was only significant in patients with macroalbuminuria. However, no differences were observed regarding eGFR loss. In the EXSCEL trial [51], eGFR was <60 mL/min/1.73 m^2^ in 17% of exenatide and placebo patients. Secondary renal endpoints were not predefined. However, post-hoc analysis demonstrated a significant reduction in the risk of a combined kidney endpoint consisting of a 40% reduction in the rate of eGFR decline, onset of dialysis or transplantation, renal death and onset of macroalbuminuria (HR 0.85 (95% CI 0.73–0.98, *p* = 0.027)) [58]. In the LEADER trial [19,52], 23% of patients had eGFR <60 mL/min/1.73 m^2^, 36% albuminuria >30 mg/g and 10% albuminuria >300 mg/g. Liraglutide decreased by 22% (HR 0.78 (95% CI 0.67–0.92, *p* = 0.003)) the risk of a secondary composite kidney endpoint (new-onset macroalbuminuria, sustained serum creatinine duplication, initiation of RRT or renal death). This benefit was mainly at the expense of macroalbuminuria reduction, without significant benefit in the other composite endpoint parameters. Interestingly, when results were stratified according to the eGFR at baseline, the decrease in eGFR in patients with a baseline estimated GFR of 30 to 59 mL/min/1.73m^2^ was 2 mL/min/1.73 m^2^ in the liraglutide group, compared with 4 mL/min/1.73 m^2^ in the placebo group (HR 1.07 (95% CI 1.04–1.10, *p* < 0.001)) [52]. In the HARMONY trial [23], eGFR was <60 mL/min/1.73 m^2^ in 11% of patients. After 1.6 years of follow-up, no significant benefit of albiglutide was observed in the safety endpoint of the eGFR decline rate. To date, no other kidney endpoints have been analyzed. In the REWIND trial [37], eGFR was <60 mL/min/1.73 m^2^ in 22% of patients. Dulaglutide achieved a significant 15% risk reduction (HR 0.85 (95% CI 0.77–0.93, *p* = 0.0004)) in the renal composite endpoint (new-onset macroalbuminuria, sustained decreased of eGFR <30% or the initiation of RRT), mainly driven by decreased new-onset macroalbuminuria. The effect of dulaglutide on renal outcome was further explored in a set of sensitivity analyses, which demonstrated that dulaglutide was associated with reduced incidence of sustained eGFR decline of 40% or more (HR 0.70 (95% CI 0.57–0.85, *p* = 0.0004)), and 50% or more (HR 0.56 (95% CI 0.41–0.76, *p* = 0.0002)). Consistent with these results, a recently published sub-analysis of another antidiabetic class drug, empagliflozin, a sodium glucose cotransporter-2 inhibitor, demonstrated that lower thresholds of eGFR (e.g., 30%) were associated with higher event rates but weaker treatment effect, suggesting that in the future renal endpoints should target an eGFR decline above 40% [59].

In the SUSTAIN 6 trial [20], eGFR < 60 mL/min/1.73 m^2^ was present in 28.5% of patients and 12.7% had baseline macroalbuminuria. Semaglutide effected a 36% reduction (HR 0.64 (95% CI 0.46–0.88, *p* = 0.005)) in a secondary combined kidney endpoint (new-onset macroalbuminuria, doubling serum creatinine reaching an eGFR <45 mL/min/1.73 m^2^, initiation of renal replacement therapy or renal death). The renal benefit owed mainly to impact on new onset macroalbuminuria (2.7% in the semaglutide group vs. 4.9% in the placebo group, *p* = 0.001). Post-hoc analyses showed a 30% reduction in albuminuria and regression to micro- or normoalbuminuria occurred for all degrees of albuminuria in patients treated with semaglutide [60]. 

In a post-hoc analysis, patients on 1 mg weekly subcutaneous dose of semaglutide had significantly slower eGFR loss than those on placebo (semaglutide 1 mg: –1.05 [–1.41; –0.69] vs. placebo: –1.92 [–2.18; –1.67] mL/min/1.73 m^2^/year, *p* < 0.001). Slower eGFR decline was also observed when patients with eGFR <60 or >60 mL/min/1.73 m^2^ were analyzed separately [60]. A recent sub-analysis confirmed a significantly milder eGFR decline with 1 mg semaglutide in patients with baseline eGFR between 30 and 60 mL/min/1.73 m^2^ [58]. In the PIONEER 6 trial [54], which evaluated oral semaglutide, 27% of patients had eGFR <60 mL/min/1.73 m^2^; however, this study did not include renal endpoints. Interestingly, in REWIND, LEADER and SUSTAIN 6, no differences were observed in RRT components of the combined kidney endpoint.

## 7. Potential Mechanisms for GLP-1RA-Associated Nephroprotection

GLP-1R have been identified in vascular smooth muscle cells of preglomerular arterioles in human and experimental animal kidneys [61] but cannot be easily detected by immunohistochemistry or imaging, and levels of expression may be below the sensitivity of the techniques [62]. Their presence in renal glomeruli and proximal tubular cells remains a subject of debate [63]. Renal expression of GLP-1R could underlie observations of GLP-1RA nephroprotection [30]. 

Indirect nephroprotective effects may depend on improvement in conventional risk factors for DKD, such as glycemic control, weight control and BP. Although nephroprotection has been observed with other glucose-lowering drugs that decrease weight such as SGLT2i, the latter possess many other potential nephroprotective mechanisms, while conferring a more modest benefit in weight reduction. Nevertheless, in the LEADER study, the difference in kidney outcome was not altered by adjustment for change in glycemic control, body weight or systolic BP [19]. 

In a meta-analysis of 26,654 patients from 33 clinical trials including 12,469 patients that received liraglutide (41%) or exenatide (59%), GLP-1RA were associated with a systolic BP reduction of 2.22 mmHg (95% CI: –2.97–1.47). In a separate meta-regression analysis, the degree of systolic BP change was not associated with baseline BP, weight loss or improvement in HbA1c [64]. Reduction in glucose levels and improvements in insulin sensitivity following therapy with GLP-1RA also lead to a decline in insulin levels, with benefits to different organs including the kidneys [39]. Treatment with GLP-1RA is able to modulate the microbiome of mice; accordingly, liraglutide could modulate the composition of the gut microbiota, leading to a more lean-related profile that was consistent with its weight-losing effect [65]. 

Direct renal and cardiac effects may also contribute to nephroprotection. The potential mechanisms of direct renal benefit of GLP-1RA are multiple and incompletely understood, most having been demonstrated in experimental animals only. These putative renoprotective actions and effects of GLP-1RA on kidneys are shown in Figure 2.

Turning next to the effect on natriuresis, GLP-1 has been demonstrated to induce natriuresis and diuresis, likely involving inhibition of sodium–hydrogen exchanger 3 (NHE3) localized at the brush border of the renal proximal tubular cells [66]. NHE3 activity in the proximal tubule increases distal tubular sodium transport in the kidney to the macula densa, resulting in restored tubular glomerular feedback with a reduction in intraglomerular pressure, hyperfiltration and renin–angiotensin system activation [67]. These findings imply that GLP-1RA are proximal diuretics and renal vasodilators that under healthy conditions only mildly influence tubuloglomerular feedback [68]. 

GLP-1RA also decrease circulating concentrations of angiotensin II, an effect that also plays a role in the observed increase in renal sodium wasting [29], and which may partially explain the BP-lowering effects of GLP-1RA. Several molecules play a role in glucose metabolism including insulin, ATP and glucose itself, which regulate NHE3 and SGLTs in the kidney, thus suggesting certain indirect natriuretic actions of GLP-1 [32]. Some studies in experimental animals have also suggested the effects of GLP-1 may be mediated by Atrial Natriuretic Peptide [39]. 

### 7.1. GLP-1RA and Renal Hemodynamics

The question of whether GLP-1RA modulate glomerular hemodynamics is controversial. GLP-1RA may decrease endothelin-1 and angiotensin II-induced vasoconstriction, thus decreasing glomerular hyperfiltration [63]. However, in healthy overweight men, exenatide infusion was found to acutely induce nitric oxide-dependent glomerular afferent arteriole vasodilation, increasing postprandial GFR by 20% (18–20 mL/min/1.73 m^2^, *p* = 0.021) [69]. Theoretically, however, decreased proximal sodium reabsorption would initiate vasoconstriction of the preglomerular arteriole through tubule-glomerular feedback. Yet, the net effect of exenatide on preglomerular arterioles was vasodilation in the present study, suggesting a stronger direct vasodilation effect. These findings imply that GLP-1RA are proximal diuretics and renal vasodilators that only mildly influence tubule-glomerular feedback under healthy conditions [68]. 

This discrepancy may be due to the fact that the renal effect of exenatide in healthy men may differ from that in insulin-resistant subjects and T2DM patients, which could explain the above-mentioned decrease in GFR after GLP-1 peptide infusion in these individuals. However, this was not replicated in patients with T2DM [69], which was shown/hypothesized to depend on impaired nitric oxide-dependent vasodilation in T2DM patients. Thus, GLP-1RA have the potential to increase or decrease eGFR, depending on baseline conditions.

The net effect GLP-1RA have on glomerular hemodynamics probably depends on the balance of the potential effect on tubule-glomerular feedback of GLP-1RA induced natriuresis (vasoconstriction of the afferent in some patients) vs. vasodilation by nitric oxide, added to the vasodilator effect of the efferent by inhibition of the renin–angiotensin axis and endothelin. The total sum of these divergent forces determines changes in the eGFR, which could explain why in LEADER [19] and SUSTAIN [20], patients presented different changes in eGFR depending on the subgroups stratified according to eGFR at baseline.

### 7.2. Modulation of Cyclic Adenosine Monophosphate–Protein Kinase a (Camp/PKA) Signaling and Other Anti-Inflammatory Pathways

GLP-1R activation leads to stimulation of cyclic adenosine monophosphate–protein kinase A pathways, producing antioxidative effects; it, therefore, seems likely that GLP-1 protects the kidney from oxidative injury [40]. In the diabetic nephropathy rat model, GLP-1RA also downregulated expression of several inflammatory biomarkers in rats, such as tubulointerstitial tumor necrosis factor alpha (TNF-α), monocyte chemoattractant protein-1(MCP-1), collagen I, alpha-smooth muscle actin (α-SMA) and fibronectin, all reported to play a role in diabetic nephropathy, as well as ameliorating kidney tubules and tubulointerstitial lesions [70]. 

Other putative renoprotective actions of GLP-1RA are the improvement of renal hypoxia related to vascular rarefaction induced by hyperglycemia and anti-atherogenic effects, both directly via an effect on glucose, lipids, weight and BP and possibly indirectly via anti-inflammatory and anti-ischemic activity [39]. 

## 8. Ongoing Studies and Unanswered Questions

There are three ongoing studies of GLP-1RA in primary kidney outcomes: Effect of GLP-1RA, liraglutide, on DKD (NCT01847313); Effect of LIXIsenatide on the Renal System (ELIXIRS) (NCT02276196); and The FLOW study (Effect of semaglutide versus placebo on the progression of renal impairment in subjects with T2DM and CKD) (NCT03819153) [71]. This last trial is the largest one designed to test the effect of once-weekly treatment with 1 mg long-acting subcutaneous semaglutide in T2DM patients with moderate/advanced CKD and albuminuria. FLOW has recently begun recruiting more than 3000 adult T2DM patients with DKD on maximal tolerated dose RAAS blockers. DKD is defined as either albuminuria 300–5000 mg/g and eGFR 50–75 mL/min/1.73 m^2^ or albuminuria 100–5000 mg/g and eGFR 25–50 mL/min/1.73 m^2^. The primary composite kidney outcome is composed of a persistent ≥50% reduction in eGFR or a persistent eGFR <15 mL/min/1.73 m^2^ or the initiation of RRT (dialysis or renal transplant) or renal or cardiovascular death. 

Several randomized clinical trial studies with GLP-1RA are currently ongoing. Among them, the Heart Disease Study of Semaglutide in Patients with T2DM (SOUL) (NCT03914326) focused on evaluating the effect of oral semaglutide in preventing cardiovascular events in T2DM patients with cardiovascular disease or CKD. In this clinical trial, the first occurrence of a composite CKD endpoint, namely renal death/onset of persistent 50% or more reduction in eGFR (CKD-EPI)/onset of persistent eGFR (CKD-EPI) below 15 mL/min/1.73 m^2^ initiation of chronic renal replacement therapy (dialysis or kidney transplantation) is included in the secondary outcomes. In addition, a new GLP-1RA named efpeglenatide is being tested in T2DM patients with established cardiovascular disease or aged over 50 years (male), 55 years (female) or older with eGFR ≥25 and <60 mL/min/1.73 m^2^ and at least one cardiovascular risk factor. In the AMPLITUDE-O trial (Effect of Efpeglenatide on Cardiovascular Outcomes) (NCT03496298), the time to first occurrence of any of the following clinical events: new-onset or progression to macroalbuminuria (>300 mg/g) accompanied by a UACR value increase of ≥30% from baseline, sustained a ≥40% decrease in eGFR from baseline (for ≥30 days), chronic dialysis (for ≥90 days), renal transplant, sustained eGFR <15 mL/min/1.73 m^2^ (for ≥30 days) is also considered as a secondary outcome.

Although several basic research studies and randomized clinical trials focused on GLP-1RA are already underway, the renoprotective mechanisms of GLP-1RA are not completely understood. At the pipeline level, further research is needed on the positive effect in terms of proteinuria, and inhibition of the sodium–hydrogen exchanger 3 (NHE3) localized at the brush border of the renal proximal tubular cells. This direct effect at the proximal renal tubular level may play a role in the differential GFR effect depending on different degrees of DKD. SGLT-2 and GLP-1RA in combination have proven effective in T2DM patients, but whether this drug combination exerts a potentiated or decreased cardiorenal protective effect in patients with moderate-advanced DKD is unknown. The threshold of human GLP-1RA is eGFR under 15 mL/min/1.73 m^2^, but this owes mainly to the lack of clinical trials in DKD patients with stage 5 CKD in dialysis programs or kidney transplantation. Thus, the safety of this class of drugs in this specific CKD population is also yet to be resolved. More studies are required in patients with advanced CKD and T2DM.

## 9. Conclusions

Recently, certain glucose-lowering drugs have shown benefits beyond blood glucose control. SGLT2i and GLP-1RA are associated with improved cardiovascular and kidney outcomes. Currently, SGLT2i has regulatory approval only in patients with eGFR >60 mL/min/1.73 m^2^, although this is expected to change soon. In contrast, human GLP-1RA can be used up to eGFR of 15 mL/min/1.73 m^2^. GLP-1RA offer the potential for adequate glycemic control in multiple stages of DKD without an increased risk of hypoglycemia and with additional benefits in weight reduction, cardiovascular outcomes and exploratory kidney outcomes. The ongoing FLOW RCT is assessing the impact of semaglutide on primary kidney outcomes in DKD.

## Figures and Tables

**Table 1:** Risk factors for diabetic kidney disease.

Risk Factor	Susceptibility	Initiation	Progression
Demographic			
Older age	**+**		
Sex (men)	**+**		
Race (black, other ethnic minorities)	**+**		**+**
Reduced renal mass	**+**		**+**
Low birth weight	**+**		
Low socioeconomic level		**+**	**+**
Hereditary			
Family history of DKD	**+**		
Genetic kidney disease		**+**	
Systemic conditions			
Hyperglycemia (poorly controlled)	**+**	**+**	**+**
Obesity	**+**	**+**	**+**
Hypertension (poorly controlled)	**+**		**+**
Kidney injuries			
Acute kidney injury		**+**	**+**
Toxins, nephrotoxic drugs, mainly NSAIDs		**+**	**+**
Smoking			**+**
Urological problems (infection, obstruction)		**+**	**+**
Dietary factors			
High protein intake	**+**		**+**

DKD: diabetic kidney disease; NSAIDs: nonsteroidal anti-inflammatory drugs. Modified from Alicic [6].

**Table 2:** Key GLP-1RA RCTs with cardiovascular and renal endpoints.

Drug (Ref)	Trial	*n*	Studied Population	Mean Duration	Composite Primary CV Endpoint	Result HR (95% CI; *p*)	Individual Primary CV Endpoint	Result HR (95% CI; *p*)
Lixisenatide [45]	ELIXA	6068	T2D and acute coronary syndrome	25 m	3P-MACE	Neutral	None	Neutral
Exenatide [46]	EXSCEL	14,752	T2D with or without CVD	3.2 y	3P-MACE	Neutral	None	Neutral
Liraglutide [19,47]	LEADER	9340	T2D and high CV risk	3.8 y	3P-MACE	0.87 (0.78–0.97; *p* < 0.001)	Death from any cause	0.85 (0.74–0.97; *p* = 0.02)
Semaglutide [20] (sc)	SUSTAIN-6	3297	T2D 50 y or more with established CVD, CHF or CKD G3 or higher or >60 y w/CV risk factor	2.1 y	3P-MACE	0.74 (0.58–0.95; *p* = 0.02)	Nonfatal stroke	0.61 (0.38–0.99; *p* = 0.04)
Albiglutide [48]	HARMONY	9469	T2D and CVD or CV risk factors	3.8 y	3P-MACE	0.78 (0.68–0.90; *p* = 0.0006)	Fatal or nonfatal myocardial infarction	0.75 (0.61–0.90, *p* = 0.003)
Dulaglutide [28]	REWIND	9901	T2D and CVD or CV risk factors	5.4 y	3P-MACE	0.88 (0.79–0.99; *p* = 0.026)	Nonfatal Stroke	0.76 (0.61–0.95; *p* = 0.017)
Semaglutide [49] (oral)	PIONEER-6	3183	T2D and CVD or CV risk factors	15.9 m	3P-MACE	Neutral	None	Neutral
Exenatide [22]	FREEDOM-CVO	4000	T2D and CV disease	UK	UK	UK	UK	UK

T2D, type 2 diabetes mellitus; CVD, Cardiovascular disease; 3P-MACE, 3-point MACE (death from cardiovascular causes, nonfatal myocardial infarction, or nonfatal stroke); SC; subcutaneous, UK, unknown; y, years; m, Month; RCTs: randomized clinical trial.

**Table 3:** Key GLP-1RA RCTs with kidney endpoints.

Drugs	Trials	% *n* eGFR < 60	Composite Kidney Endpoint	Results	Individual Kidney Endpoint	Result HR (95% CI; *p*)
Lixisenatide [45]	ELIXA	23	NA	NA	New onset macroalbuminuria	0.808 (0.660–0.991; *p* = 0.0404)
Exenatide [46]	EXSCEL	17	40% reduction in eGFR loss, onset of dialysis or transplantation, renal death and onset of macroalbuminuria	0.85 (0.73–0.98; *p* = 0.027)	None	Neutral
Liraglutide [19,47]	LEADER	23	New onset macroalbuminuria, sustained serum creatinine duplication, initiation of renal replacement therapy or renal death	0.78 (0.67–0.92; *p* = 0.003)	New onset macroalbuminuria	0.74 (0.37–0.77; *p* = 0.001)
Semaglutide [20] (sc)	SUSTAIN-6	28.5	New onset macroalbuminuria, doubling serum creatinine reaching an eGFR <45 mL/min/1.73 m^2^, initiation of renal replacement therapy or renal death	0.64 (0.46–0.88; *p* = 0.005)	Persistent macroalbuminuria	0.54 (0.60–0.91; *p* = 0.004)
Albiglutide [48]	HARMONY	11	UK	UK	UK	UK
Dulaglutide [28]	REWIND	22	New onset macroalbuminuria, sustained decreased of eGFR <30% or the initiation of renal replacement therapy	0.85 (0.77–0.93, *p* = 0.0004)	New onset macroalbuminuria; Sustained decline in eGFR of ≥40%; Sustained decline in eGFR of ≥50%	0.77 (0.68–0.87; *p* < 0.0001); 0.70 (0.57–0.85; *p* = 0.0004); 0.74 (0.66–0.84; *p* < 0.0001)
Semaglutide [49] (oral)	PIONEER-6	27	UK	UK	UK	UK
Exenatide [22]	FREEDOM-CVO	UK	UK	UK	UK	UK

NA, not apply; SC; subcutaneous; UK, unknown; RCTs: randomized clinical trial.
